# Myelomeningocele closure: A review and decision-making guidance

**DOI:** 10.1016/j.jpra.2025.10.005

**Published:** 2025-10-16

**Authors:** Elie Ghadban, Maria Zouein, Jad Sleiman, Karl Andary, Samer Bassilios Habre

**Affiliations:** a Division of Plastic and Reconstructive Surgery, Saint George University of Beirut Achrafieh, Beirut, Lebanon; b Saint George University of Beirut Achrafieh, Faculty of Medicine, Beirut, Lebanon

**Keywords:** Myelomeningocele, Myelomeningocele repair, Primary closure, Fasciocutaneous flap, Musculocutaneous flap

## Abstract

**Background:**

Myelomeningocele, the most severe neural tube defect, results from failed neural tube closure during embryogenesis, leading to herniation of the spinal cord and meninges through a vertebral defect. Early postnatal surgical intervention remains the standard of care to prevent infection, preserve neurological function, and achieve durable soft tissue coverage. Despite advances in reconstructive techniques, wound complications remain common, and consensus on the optimal technique and flap selection remains inconclusive.

**Objective:**

This review examines current evidence on postnatal myelomeningocele closure techniques, including flap-based reconstructive methods, aiming to guide flap selection based on defect characteristics.

**Results:**

Primary skin closure remains suitable for small defects (<20–25 cm² or width <5 cm), offering low morbidity but variable complication rates, particularly in larger lesions. Skin grafting, once commonly used, is now largely abandoned due to its poor long-term durability and tendency to induce kyphotic deformities. Local fasciocutaneous flaps, including Limberg rhomboid, V-Y advancement, keystone perforator island flaps, and the butterfly flap, demonstrate reliable outcomes in moderate to large defects, with low rates of cerebrospinal fluid leak, dehiscence, and flap necrosis. More extensive defects may necessitate musculocutaneous flaps (e.g., paraspinous, latissimus dorsi, gluteus maximus), which offer solid coverage but are associated with increased operative morbidity and potential functional impairment.

**Conclusion:**

Optimal myelomeningocele reconstruction requires individualized surgical planning guided by defect-specific parameters and surgical expertise. Flap selection should prioritize tension-free closure, minimal donor-site morbidity, and durability. This review proposes an algorithmic, anatomy-informed approach to assist clinicians in achieving favorable outcomes across a wide spectrum of myelomeningocele presentations.

## Introduction

Myelomeningocele (MMC), the most severe and dreaded form of neural tube defects, is a type of open spinal dysraphism or neural tube defect resulting from improper closure of the neural tube that usually occurs at week four of pregnancy, leading to herniation of a part of the spinal cord and meninges through the vertebral defect. The etiology of myelomeningocele is often multifactorial, involving environmental, maternal, and genetic factors.^1^ Globally, an estimated 214,000 to 322,000 cases of neural tube defects occur annually, with an overall prevalence of 3.1 cases per 10,000 births in the United States.^2^

Prenatal closure can be considered as a treatment option. Yet, despite the potential benefits of fetal surgery, this technique remains limited to select specialized centers, as it is technically demanding and requires extensive multidisciplinary collaboration and high expertise.^3^^,^^4^ Consequently, postnatal closure remains the standard of care. It typically occurs as early as 24 to 48 h after birth, in order to protect exposed neural tissues and reduce the risk of infection.^5^ Prior to the 1960s, surgical repair was often delayed, and results were poor due to complications such as meningitis and hydrocephalus for which the occurrence was common at the time and resulted in a decline of the survival rate to <12 %.^6^ However, with the introduction of antibiotics and cerebrospinal fluid (CSF) shunting in the late 1950s, along with a shift toward rapid surgical intervention, survival and long-term outcomes dramatically improved.^6^

Advancements in reconstructive surgical techniques have now made it possible to effectively close even large defects, achieving durable coverage with tissue flaps.^3^ Despite this, wound complications remain a challenge, and the literature lacks a clear, standardized approach to flap selection. In this context, this review aims to comprehensively examine postnatal myelomeningocele closure techniques, with a particular emphasis on flap-based reconstructive methods. More in-depth, it seeks to look further into surgical indications, evaluate the clinical outcomes and limitations of different flap techniques, and provide evidence-based guidance with an algorithmic approach for choosing appropriate flap methods tailored to individual defect characteristics.

## Methods

A comprehensive search of the PubMed database was conducted using the keywords “myelomeningocele,” “surgical repair,” “postnatal closure,” “flap techniques,” “skin grafting,” “musculocutaneous flaps,” and “fasciocutaneous flaps” from inception until 2025. Reference lists of relevant articles were also reviewed to identify additional sources.

Studies included in this review focused on specific flap techniques, clinical outcomes, and complications. Emphasis was placed on comparing flap techniques and their respective outcomes. The inclusion criteria focused on English-language articles that discussed postnatal myelomeningocele closure, specifically those addressing clinical outcomes related to flap reconstruction techniques. Studies were required to report on outcomes such as CSF leakage, infection rates, wound dehiscence, and long-term stability, and must have involved human subjects. Studies were excluded if they focused solely on prenatal closure, were unrelated to myelomeningocele pathology, or lacked sufficient clinical data.

The evidence was primarily drawn from retrospective case series and comparative analyses. Data was synthesized qualitatively. Although no formal grading of evidence was applied, methodological limitations that may affect the validity and generalizability of the findings were noted and discussed.

## Anatomy and embryology

Initially, the ectoderm, the outermost germ layer in the early embryo, which mainly gives rise to the central nervous system and skin, differentiates into the neuroectoderm and forms the neural plate at approximately the third week of embryonic life. Its lateral edges later become elevated and form the neural folds, which fuse together to form the neural tube starting from the cervical region in both directions: cranially and caudally. This process is known as primary neurulation, and failure of the closure of the neural tube by the 4th week of development gives rise to neural tube defects.^7^

Myelomeningocele represents the most severe and clinically significant form of spina bifida, arising from a critical defect in early embryonic development as a result of failure of primary neurulation.^2^^,^^8^^,^^9^ During this period, the neural plate undergoes a complex sequence of folding and fusion events to form the neural tube, the precursor to the central nervous system. In MMC, the caudal portion of the neural tube fails to close properly, leading to a persistent midline defect in the vertebral column.^2^^,^^8^, ^9^, ^10^

This failure in neural tube closure allows portions of the spinal cord, meninges, and associated neural elements to herniate through the vertebral defect, forming an external sac or lesion, the neural placode. The latter typically lies flush with the skin and is often adherent to the margins of the open defect. In many cases, the placode is incompletely covered by a meningeal sac, and in severe presentations, may lack any protective covering altogether.^2^^,^^8^, ^9^, ^10^ This anatomical exposure renders the developing neural tissue susceptible to mechanical and chemical injury from the intrauterine environment, particularly from amniotic fluid.^2^^,^^8^, ^9^, ^10^ Histological features of MMC include disorganized spinal cord structure, malformed or absent nerve roots, and central canal dilation.^11^

## Classification

Given the wide variability in myelomeningocele defect size, location, and associated spinal anomalies, creating a standardized classification system for surgical repair remains complex. Most proposed algorithms are based on clinical experience and anatomical perspectives. Some of the most widely referenced classification systems in the literature are depicted in Table 1.

**Table 1:** Classification systems for surgical closure of myelomeningocele repair.

Study	Basis for classification	Classification
Kankaya et al.^12^	Defect transverse diameter-to-back width ratio or defect area-to-donor area ratio	<0.30 or <8 % → Primary closure
		0.30–0.50 or 8–15 % → 2 V-Y rotation flaps if no kyphosis; advanced technique if kyphosis
		>0.50 or >15 % → 4 V-Y (butterfly) flap with kyphosis consideration
Kemaloglu et al.^13^	Defect height (y)/width (x) ratio and posterior axillary line/defect width ratio	y/*x* < 1.5 → Flap closure
		y/*x* = 1.5 → Use posterior axillary line/defect width ratio: if ≥3 → primary closure; if <3 → flap closure
Sharma et al.^14^	Defect size and shape	<7 cm² with tension-free margins → Direct repair
		>7 cm² → Flap closure
		Circular defects → Double/triple rotation flaps
		Rhomboid defects → Limberg flap
		Square/triangular defects → Local transposition flap
Putri et al.^15^	Defect size and defect-to-back width ratio	<15 cm² or <0.40 ratio → Type II keystone flap
		15–30 cm² or 0.40–0.69 → Type III or unilateral Type IV keystone flap
		>30 cm² or >0.69 → Bilateral Type IV keystone flap
Algan et al.^16^	Defect area	<25 cm² → Unilateral Limberg flap
		25–35 cm² → Bilateral Limberg flap
		>35 cm² → Bilateral bipedicled advancement flap
Fairchild et al.^17^	Defect size	<5 cm → Primary transverse closure
		>5 cm → Closure based on location: thoracic, lumbar/lumbosacral, or sacral. Tailored strategy depending on regional anatomy and defect extent

In 2015, Kankaya et al. proposed an algorithm grounded in the defect’s transverse diameter-to-back width ratio or the defect area-to-donor area ratio. For values <0.30 or <8 %, primary closure was deemed appropriate regardless of kyphosis. When the ratio fell between 0.30–0.50 or 8–15 %, the presence of kyphosis determined the approach: patients without kyphosis received a two V-Y rotation-advancement flap, while those with kyphosis required more complex reconstruction. For ratios >0.50 or 15 %, a four V-Y rotation-advancement (butterfly) flap was recommended.^12^

Kemaloğlu et al. later introduced a different closure algorithm based on the defect height (y)-to-width (x) ratio and the thoracic width-to-defect width ratio. If y/*x* ≥ 1.5, the ratio of the length between the posterior axillary lines to defect width determines the technique: a ratio equal to or >3 calls for primary closure, while a ratio <3 suggests flap usage. If y/*x* < 1.5, flap closure is recommended. Surgeries were performed by two surgeons using either primary or Limberg/bipedicled flaps. Patients were one to five days old, all had lower limb paralysis, and 20 % of them had additional anomalies. Mean follow-up was 6.8 years.^13^

In 2019, Sharma et al. introduced an algorithm based on defect size and geometric configuration. Defects ≤7 cm² with approximable skin margins were closed primarily. Larger defects potentially require flap reconstruction, with flap selection guided by shape. Notably, circular defects were managed with double or triple rotation flaps, rhomboid defects with Limberg flaps, and square or triangular defects with local transposition flaps.^14^

In 2021, Putri et al. advanced a more developed classification system, integrating both defect size and defect-to-back width ratio. Defects with a surface area of <15 cm² or with a width ratio of <0.40 were managed using a type II keystone flap. For defects with a surface area ranging from 15 to 30 cm² or with ratios between 0.40 and 0.69, a type III or unilateral type IV keystone flap was indicated, while defects exceeding an area of 30 cm² or with a width ratio of >0.69 warranted a bilateral type IV keystone flap. This approach resulted in tension-free closure with optimal skin quality and operative efficiency, as described by the authors.^15^

Alternatively, Algan et al. classified the MMC repair techniques based on absolute defect size, reviewing 45 cases treated with various fasciocutaneous techniques. Their algorithm recommended unilateral Limberg flaps for defects <25 cm², bilateral Limberg flaps for those between 25–35 cm², and bilateral bipedicled advancement flaps for defects exceeding 35 cm². Despite instances of wound dehiscence and partial necrosis, all cases achieved successful healing with conservative management, demonstrating the adaptability of the Limberg flap within a size-stratified reconstructive framework.^16^

More recently, Fairchild et al. proposed a structured approach beginning with dural repair and lumbar paraspinous muscle closure. After excision of atrophied skin, transverse defect size guided further intervention. Defects <5 cm were closed with a primary transverse technique. For larger defects measuring >5 cm, closure strategy was dictated by anatomical location, including thoracic, lumbar/lumbosacral, or sacral locations.^17^

## Principles of surgical treatment

### Dural repair

The surgical management of MMC centers on early, meticulous repair to preserve neural function, prevent infection, and restore anatomical barriers.^18^^,^^19^ The neurosurgical technique involves precise dissection of the placode along the junctional zone to preserve viable neural elements. When feasible, the pia-arachnoid edges are approximated to simulate primary neurulation, followed by a watertight dural closure to reduce the risk of meningitis.^18^ Its borders are sutured under the microscope with a 7–0 monofilament, transforming it into a structure like the primitive neural tube, which will later facilitate dural closure.^20^

Dural grafts may be required in cases with bulky placodes or a flattened spinal canal to avoid neural compression. Hydrocephalus, present in up to 85–90 % of MMC cases, is often treated with ventriculoperitoneal shunting. Although simultaneous repair and shunting can relieve intracranial pressure and promote wound healing if done within the first 48 h of life,^21^ the increased infection risk has led many centers to prefer staged procedures unless urgent CSF diversion is required.^18^^,^^19^^,^^22^^,^^23^ Early intervention, ideally within 6 to 12 h after birth, significantly reduces the risk of ventriculitis and optimizes neurological outcomes. The surgical prognosis has improved significantly with modern techniques, particularly when repair is performed early and without complications.^18^^,^^24^

### Soft tissue coverage

Soft tissue coverage is equally critical to achieving a durable and infection-resistant repair. A range of soft tissue reconstruction techniques may be used, including primary closure, split-thickness grafting, and the use of various local or regional flap closures.^4^^,^^25^ Preoperative evaluation of skin availability guides reconstructive planning, particularly in large or high-lumbar defects where primary closure may not be feasible.^26^ Redundant skin, when present, should be preserved until the neural and dural layers are secured to ensure a tension-free, layered closure.^18^

The subcutaneous layer is thin at the border of the malformation but becomes thicker at the junctional zone. Therefore, subcutaneous sutures are placed there to ensure tension-free closure. With larger defects, these sutures can be placed 2–3 cm away from the skin edge as “stay sutures” that will be tied to the underlying fascia and ensure an easy subsequent skin closure.^27^

Following this stage, the selection of the appropriate skin closure technique is carried out, representing one of the most important steps of MMC repair and the primary focus of the present review. This decision is influenced by a range of clinical and anatomical factors, which will be examined in detail in the subsequent sections.

## Surgical techniques for skin closure

### Primary skin closure

Primary closure is the simplest and most commonly used technique for small myelomeningocele defects, used immediately following neurosurgical repair to cover the dural closure, offering the benefits of a single-stage procedure, reduced operative time, and minimal morbidity.^18^

The skin is inspected for primary closure once the dural layer and soft tissue have been closed. It can be dissected bluntly (digital dissection) to free the skin from surrounding tissues in all directions. Optimal estimates of skin edges should be planned and secured with skin hooks or temporary stitches before performing a midline closure, typically using 3–0 Vicryl for subcutaneous layers and running 4–0 Prolene for the skin. For larger defects, stay sutures anchored to fascia may assist closure. Skin blanching may occur from tension but usually resolves; nitroglycerin may aid perfusion when needed.^28^

Post-surgical care includes applying an occlusive dressing to protect the wound and positioning the neonate prone with slight back elevation to reduce pressure on the closure and prevent CSF leakage.^18^

### Skin grafting

Skin grafting in myelomeningocele repair has historically been considered when primary closure is not achievable, particularly for defects larger than 20–25 cm². Split-thickness skin grafts, originally proposed by Mustardé in the 1960s, are most frequently employed, harvested from areas such as the buttocks and thigh.^29^ These grafts, often meshed at ratios like 1.5:1,^30^ are secured using interrupted or running sutures tied over a bolster dressing (cotton wool or sponge) to ensure optimal adherence and immobilization.^29^ Luce and Walsh later supported the technique either as a delayed approach or concurrently with dural closure.^30^ Full-thickness skin grafts as a sole method are rarely used in the treatment of MMC, resulting in scarcity of available literature.

Nevertheless, due to the high rates of complications, the use of skin grafting has been reserved as an adjunct to more complex flap surgeries.^31^

### Local fasciocutaneous flaps

#### Limberg rhomboid flap

The Limberg rhomboid flap involves designing a symmetrical rhombus around the defect, with four equal sides and alternating angles of 60° and 120° A full-thickness incision is placed at one 120° angle, and careful subfascial dissection preserves the perforator vessels. The flap, consisting of skin and subcutaneous tissues, is then transposed into the defect, allowing primary closure of the donor site under minimal tension.^18^^,^^32^^,^^33^

#### Z-advancement rotation flap

The Z-advancement rotation flap similarly begins with marking a rhomboidal configuration around the defect, without further excision of the defect margin. Curvilinear incisions extend laterally from the widest angles toward the gluteal and thoracic regions, surpassing the rhomboidal length by approximately 25 %. Dissection continues deep to the latissimus dorsi muscle and thoracolumbar fascia, carefully preserving underlying perforators. Subsequently, the flaps are advanced into the defect, and layered closure is performed.^34^^,^^35^

#### Z-plasty triangle closure

The Z-plasty triangle closure starts by converting the defect into a triangular form. Geometric landmarks are established in an acute triangular fashion, extending lines outwardly to form a characteristic unequal Z-plasty design, typically with 60° and 45° angles. Flaps can be oriented either horizontally or vertically, with flap perfusion assessed intraoperatively, commonly via Doppler imaging. After meticulous elevation, one flap primarily covers the defect, and the second flap addresses the donor site of the first flap, achieving tension-free closure with standard layered suturing using 4–0 Vicryl for the subcutaneous layer and 5–0 Polypropylene for the skin.^36^

#### Reading man procedure

The Reading Man Procedure also uses a Z-plasty principle, initiating from a central limb through the midline of the defect, drawn twice as long as the defect diameter, with lateral limbs positioned at angles of 60° and 45° Following careful fasciocutaneous flap dissection with preservation of perforators, one flap is transposed centrally to cover the defect, and the second flap closes the donor area, mirroring the technique of traditional Z-plasty.^37^

#### V-Y advancement flap

The V-Y advancement flap approach, also known as the butterfly flap in bilateral applications, involves outlining a rectangular area around the defect extending from the bilateral posterior axillary lines cranially to the intergluteal region caudally. The flap limbs are arc-shaped to increase distal edge surface area. The vascular supply of the flap originates from its deeper surface via paraspinous perforators; therefore, dissection is minimal at the edges and avoided in the central portion of the flap, resembling the technique used in Keystone flap dissection. Meticulous dissection includes the latissimus dorsi and thoracolumbar fascia, with careful preservation of paraspinous perforators. Final closure is executed in a double-layer closure with 4–0 Polyglactin and 5–0 Poliglecaprone 25 sutures, and the donor-site closure is performed using the classic V-Y manner.^38^^,^^39^

#### O-S closure

The O-S closure technique transforms a circular (O-shaped) defect into an S-shaped closure. Flaps are designed as opposing arcs superiorly toward the trapezius and latissimus dorsi muscles and inferiorly toward the gluteus maximus muscle and sacrum. Dissection extends laterally toward the posterior axillary folds, carefully preserving large perforating vessels. Flaps are centrally advanced, resulting in an S-shaped scar. The deep subcutaneous layer is closed with 5–0 Monocryl sutures, and the skin is finally closed with 5–0 monofilament sutures.^40^

#### Keystone-designed perforator island flap (KDPIF)

Lastly, the KDPIF features a curvilinear, trapezoidal-shaped flap designed around identified perforating vessels, which are located preoperatively via Doppler imaging. Depending on defect complexity, flap types range from simple skin advancement (Type I) to more extensive subfascial mobilization (Types II-IV), following the classification described by Behan. Type I is appropriate for defects up to 2 cm and preserves the deep fascia. Type IIA involves division of the deep fascia along the curvilinear edge to enhance tissue mobilization, while Type IIB similarly divides the fascia but incorporates a skin graft to manage secondary defects. Type III includes two opposing keystone flaps for closure of larger or more complex wounds, and Type IV features subfascial flap elevation and up to 50 % rotation.^41^^,^^42^ The flap dimensions match the defect width (with a 1:1 ratio), and flap advancement can follow either the conventional keystone or modified omega design. Bilateral flaps may also be designed for larger defects, with flap orientation tailored either cranio-caudally or laterally, depending upon defect geometry. Closure typically follows a central, tension-minimizing approach.^15^^,^^43^

### Musculocutaneous (myocutaneous) flaps

The main muscles that can be used in the myocutaneous flaps are the paraspinous, latissimus dorsi, and gluteus maximus, or a combination, typically to cover larger defects.^44^^,^^45^

#### Paraspinous musculocutaneous flap

The paraspinous musculocutaneous flap involves the dissection of the paraspinous muscles along with the thoracolumbar fascia, latissimus dorsi, and gluteus maximus fascia. This composite flap is elevated carefully, ensuring the preservation of arterial perforators and avoiding injury to surrounding structures such as the retroperitoneum. The flap is advanced medially to cover the dural repair, with skin closure performed over the midline. This technique results in a two-layer closure: a paraspinous muscle flap and a thoracolumbar fascial flap.^46^

#### Latissimus dorsi myocutaneous flap (LDMF)

The LDMF is based on the thoracodorsal artery and can be designed in various configurations, including proximal-based, distally-based, or bilateral bi-pedicled, depending on the defect's location and size. For proximal-based flaps, typically used for high-lying defects, two triangular skin islands are designed on each side of the defect, with the base of the triangles extending toward the posterior axillary line. Dissection begins on the thoracolumbar fascia, which is separated from the paraspinous muscles, and the flap is elevated, with the pedicle based on the thoracodorsal artery. The flap is advanced toward the midline, and the donor site is closed using V-Y advancement and split-thickness skin grafting.^47^

In the case of distally-based flaps, typically for defects located below the iliac crest, a skin-muscle island pedicle is elevated, incorporating perforators from the posterior intercostal arteries. The flap is mobilized and advanced toward the defect, and the donor site is closed primarily.^47^

For bilateral bi-pedicled flaps, which are used for very large defects, the latissimus dorsi muscle is carefully dissected from its attachments to preserve its blood supply. Both flaps are elevated and advanced toward the midline, where they are sutured together. The donor sites are closed with split-thickness skin grafts.^47^

#### Gluteus maximus myocutaneous flap

The gluteus maximus myocutaneous flap is particularly useful for covering lumbosacral defects. After identifying the superior gluteal artery perforators using Doppler ultrasound, the flap is carefully dissected, preserving the neurovascular supply. The flap is rotated to cover the defect, and the skin is closed in layers. The pedicled flap is stabilized at the site, ensuring good vascular support for optimal healing. Postoperatively, infants are placed in a prone position to ensure proper healing of the flap and surgical site.^48^ Note should be taken when handling the superior gluteal artery perforators to reduce potential vascular injury, since they measure approximately 1 to 2 mm in diameter in newborns.^49^

## Results

The current literature reflects a substantial evolution in the surgical management of MMC, transitioning from primary skin approximation with high complication rates to more sophisticated, multidisciplinary approaches using tension-free, well-vascularized flap techniques.

### Primary skin closure

Primary closure remains the most frequently applied method for small defects due to its simplicity, shorter operative time, and low morbidity, with multiple studies identifying cutoff points at 18 cm²,^32^ 20 cm²,^44^ 25 cm²,^18^ and even up to 40 cm²,^50^ though complication rates increase beyond these thresholds. Others, like Fairchild and Kemaloğlu^13^^,^^44^ have suggested defect width of <5 cm as a critical determinant. However, outcomes remain variable, with wound dehiscence rates ranging from minimal^13^^,^^44^ to 100 %,^51^ influenced by defect characteristics, timing of surgery, and criteria for defining complications, particularly regarding the difference between minor dehiscence and major wound breakdown.^17^^,^^44^

### Skin grafting

Given its poor long-term durability and the risk of contributing to severe kyphotic deformities, skin grafting is no longer considered a functional or reliable method of coverage in most cases, and this has led to shifts in reconstructive strategies toward more complex options including fasciocutaneous and myocutaneous flaps.^30^^,^^40^

### Local fasciocutaneous flaps

When primary closure is not feasible, local fasciocutaneous flaps provide effective alternatives. The Limberg rhomboid flap is widely used for moderate to large MMC defects, with reported success in defects up to 72 cm². While generally reliable, delayed presentations have been associated with an increased risk of flap complications due to placode tethering and epithelialization.^14^ Despite long-term occasional occurrences of necrosis and dehiscence requiring secondary interventions, these are typically managed conservatively and lead to complete healing without further complications.^4^^,^^14^^,^^33^ The Limberg flap has demonstrated consistent utility, with operative times averaging around one hour.^4^^,^^14^^,^^33^ In an attempt to compare the Limberg flap with rotation flaps, Sharma et al. noted that the latter offered similar clinical efficacy with slightly longer operative times.^14^ Moreover, Brown et al. reported excellent clinical outcomes over ninety-seven patients, reflecting that flap choice did not affect complication rates, though rhomboid flaps were associated with higher odds of minor complications.^52^

V-Y advancement flaps have been consistently associated with superior outcomes, as demonstrated in prospective and retrospective studies, showing minimal CSF leakage and wound dehiscence or infection.^38^^,^^51^^,^^53^ A comparative study by Cehan et al., evaluating primary closure, Limberg flap, and V-Y advancement flaps showed the V-Y technique as having the lowest complication rate, while the Limberg group experienced high rates of wound dehiscence and CSF fistula.^54^ Long-term follow-up of V-Y advancement flaps has also indicated durable results with low recurrence of complications, further supporting its efficacy for defect closure.^38^^,^^51^^,^^53^

More innovative techniques, such as the butterfly (quadruple V-Y) flap, binary flap, and Z-advancement-rotation (ZAR), flap have emerged for large and complex thoracolumbar defects. Kankaya and Rankin reported reliable coverage with low complication rates using the butterfly technique,^12^ while Gümüş’s ZAR flap offered a rapid, low-morbidity option with no observed complications.^35^ Emsen’s O-S closure, providing both functional and aesthetic advantages on the long run, also demonstrated successful closure in large defects without flap ischemia.^40^ Long-term outcomes of these techniques have demonstrated good functional results, though some cases report minor complications, such as partial necrosis and aesthetic concerns, which resolved with secondary healing or minor interventions.^12^^,^^35^

The triangular closure and the “Reading Man” procedures, introduced by Mutaf, were effective in moderate-sized defects, achieving tension-free closure with minimal complications and good long-term healing, although case numbers were small.^36^^,^^37^

KDPIFs, particularly Types III and IV, have proven effective for large myelomeningocele defects, with studies reporting no major complications such as flap loss or CSF leak in large defects. Across multiple series, minor issues like small dehiscence or wound infections were infrequent and managed conservatively.^15^^,^^55^, ^56^, ^57^ Unilateral flaps were associated with shorter operative times and fewer complications.^15^ Notably, Formentin et al. observed maintained flap viability even postmortem in a neonate who died from sepsis, demonstrating its reliability even under systemic compromise and for longer terms.^55^ Long-term follow-up has demonstrated excellent functional outcomes, with most patients achieving complete healing, though minor complications such as wound dehiscence were noted in some cases, which healed without affecting overall recovery.^15^^,^^55^

### Musculocutaneous (Myocutaneous) flaps

Musculocutaneous flaps, while offering durable coverage, remain controversial due to increased intraoperative morbidity.^50^ Early pioneers like Mustardé, McCraw et al., and McDevitt et al. demonstrated successful outcomes using latissimus dorsi and gluteal musculocutaneous flaps.^29^^,^^58^^,^^59^ Bagłaj et al. showed reliable outcomes in 35 neonates with defects of >50 cm² using bilateral bi-pedicled musculocutaneous flaps.^60^ The MI series by Lien et al., advocating a muscle-plus-fascia approach for all defects, reported no CSF leaks and minimal flap-related complications.^45^ Yet, other studies have noted increased bleeding, loss of muscle function, and longer operative times, making fasciocutaneous alternatives more favorable in many cases.^12^^,^^40^^,^^50^ Kobraei et al. found no statistical difference in complication rates between the two approaches.^44^ A novel modification involving adipofascial flaps with acellular dermal matrix, studied by Pourtaheri et al., showed comparable safety to myocutaneous flaps while reducing donor-site morbidity and operative complexity.^61^ Long-term follow-up of musculocutaneous flap reconstruction has shown positive functional outcomes, particularly in maintaining motor function, though aesthetic concerns such as scar formation and donor-site morbidity, especially in the shoulder or gluteal areas, remain a significant issue.^29^^,^^58^, ^59^, ^60^, ^61^

## Algorithmic approach

Nevertheless, no specific approach is seemingly ideal, but there is a general consensus in the literature on a few points. First, tension-free closure is paramount for success; thus, any method that achieves it is ultimately favored. Second, large defects often require advanced techniques including fasciocutaneous and/or muscle flaps, and have a higher baseline risk of complications simply due to size. Finally, no single technique is superior for all cases. Instead, a tailored and case-by-case approach yields the best outcomes.

## Limitations

Being a narrative review, this study is limited by the absence of a systematic search strategy, formal quality assessment, and quantitative synthesis. Additionally, it provides a broad overview of current reconstructive techniques for myelomeningocele defects, therefore the potential for selection and interpretation bias should be acknowledged.

## Conclusion

**Figure 1:**
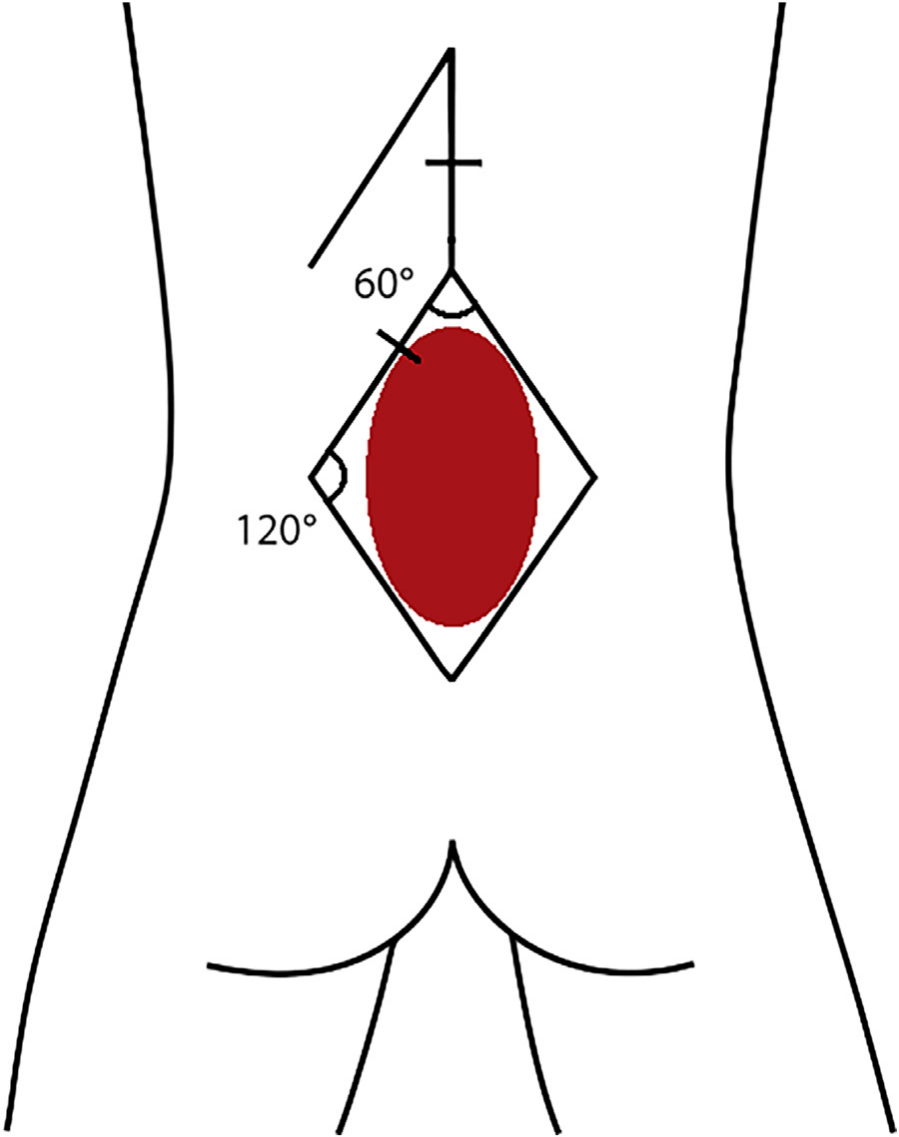
Limberg flap schematic for myelomeningocele defect closure, showing 60° and 120° angles with flap design lines for transposition.

**Figure 2:**
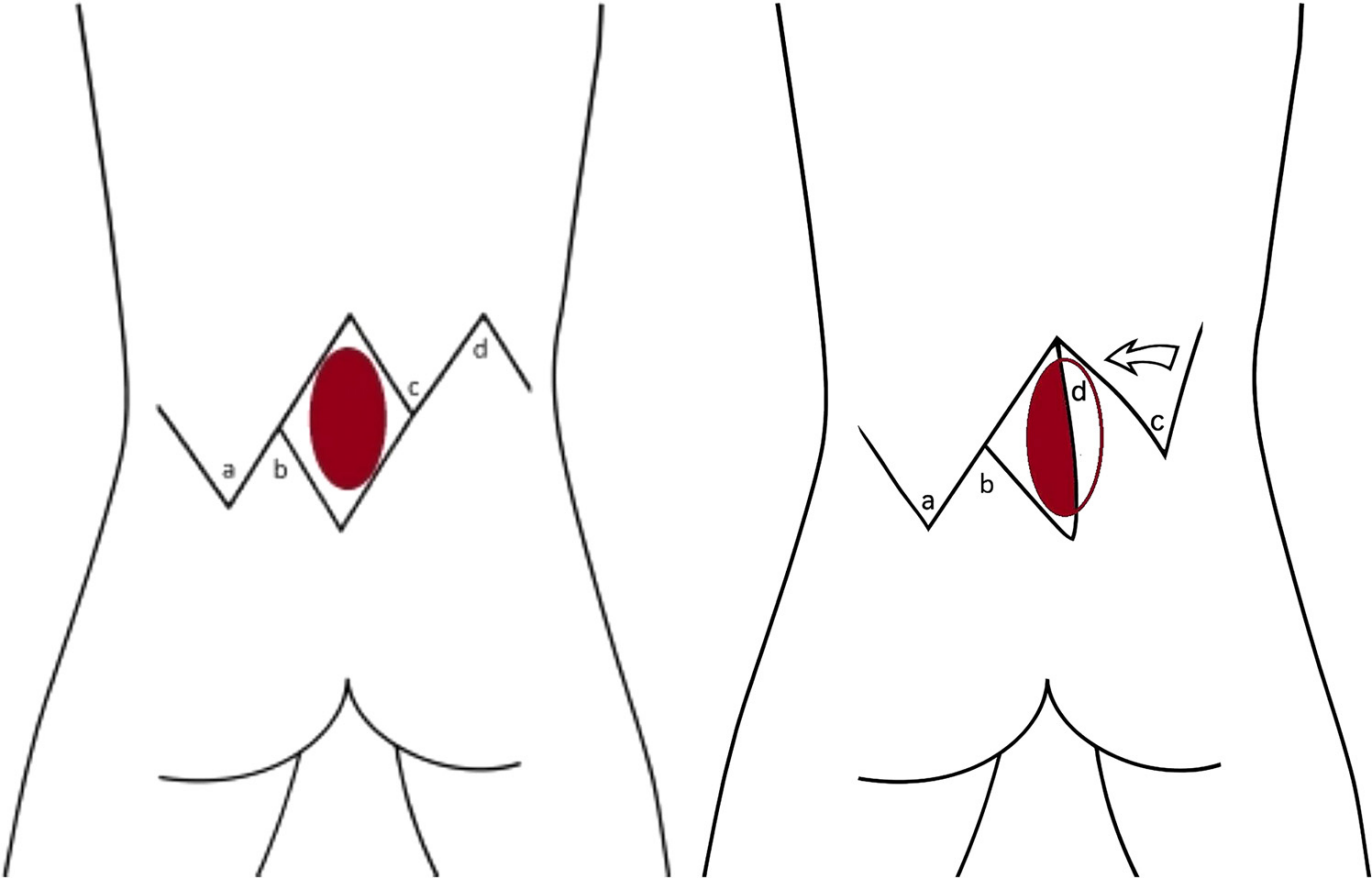
Z-advancement rotation flap schematic for myelomeningocele defect closure, showing rhomboid marking, lateral curvilinear incisions, and flap advancement into the defect for layered closure.

**Figure 3:**
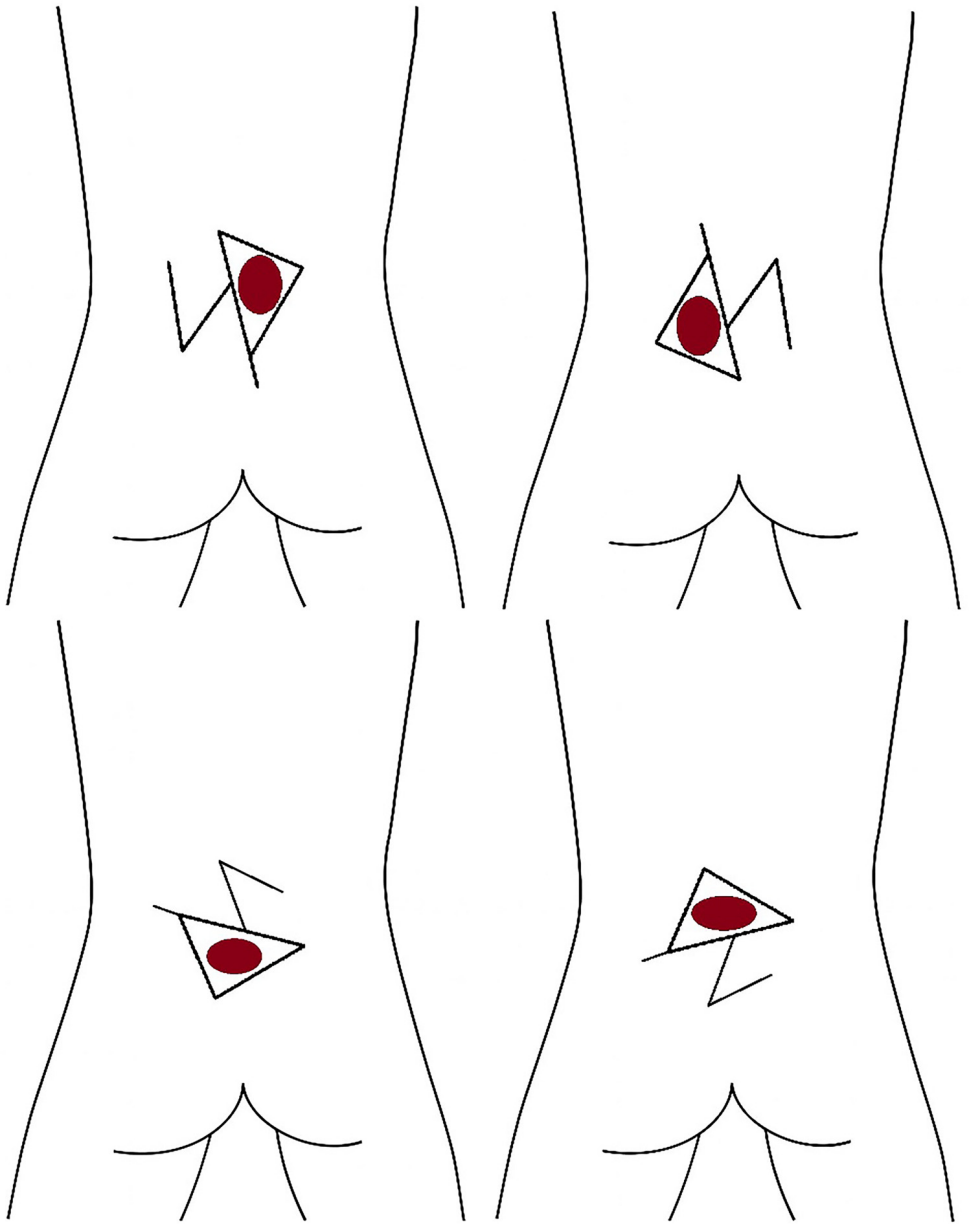
Z-plasty flap schematic for myelomeningocele defect closure, showing various applications of the technique in different orientations and flap advancements.

**Figure 4:**
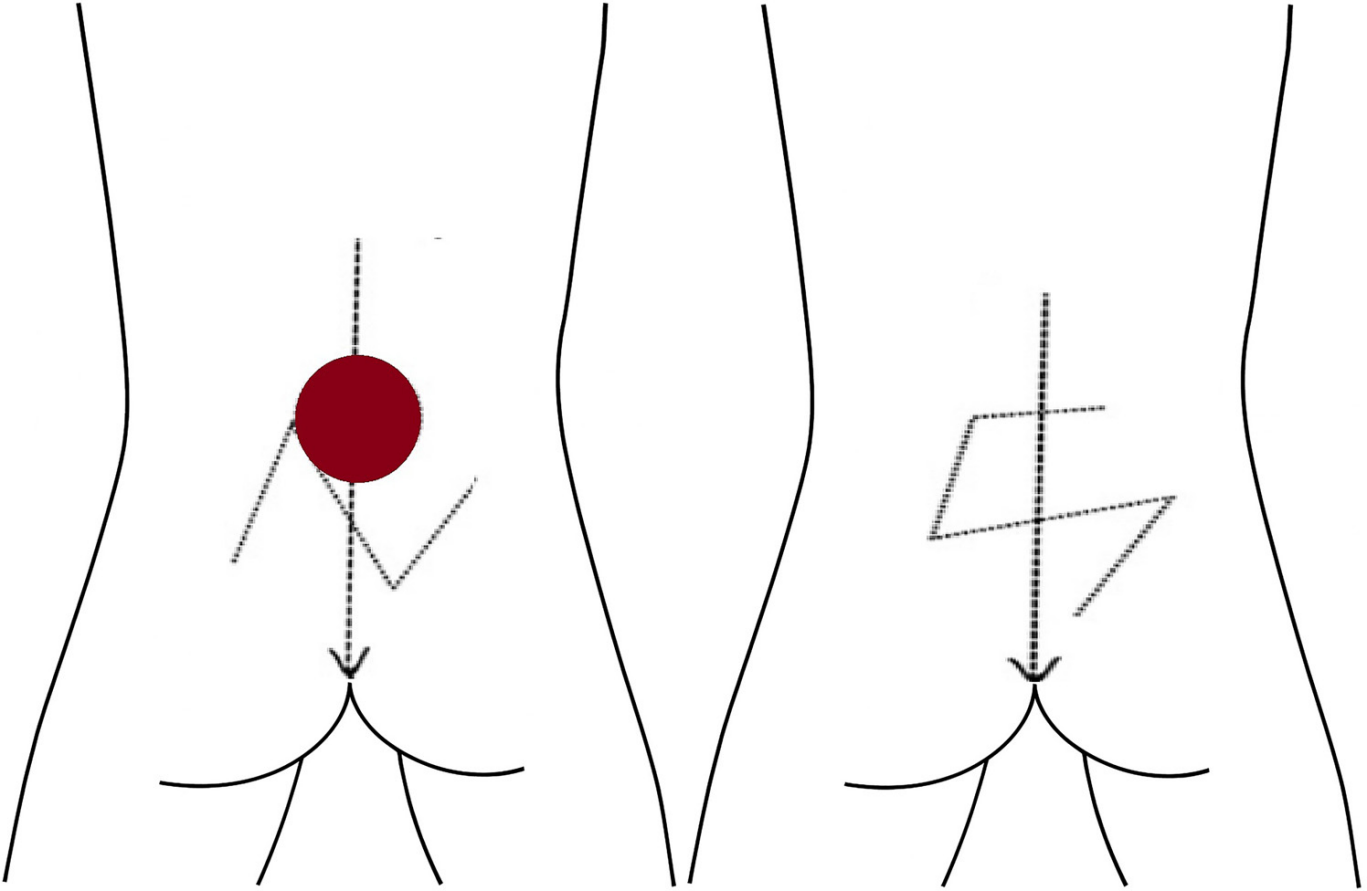
Reading Man flap schematic for myelomeningocele defect closure, showing central limb marking and flap transposition to cover the defect and close the donor site.

**Figure 5:**
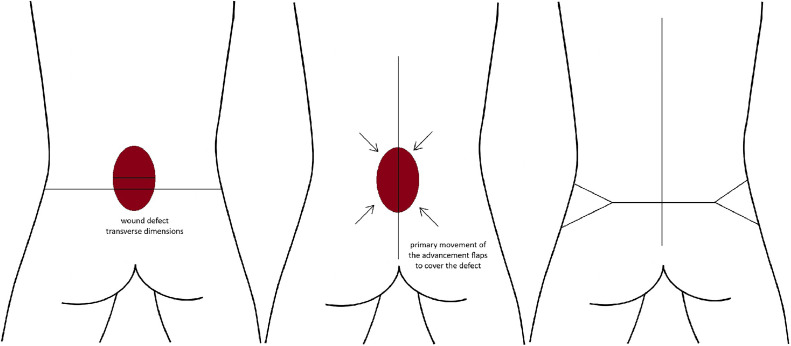
V-Y advancement flap schematic for myelomeningocele defect closure, showing sequential steps of flap design and closure. The third panel illustrates the releasing incision and the bilateral scars resulting from V-Y advancement after final closure.

**Figure 6:**
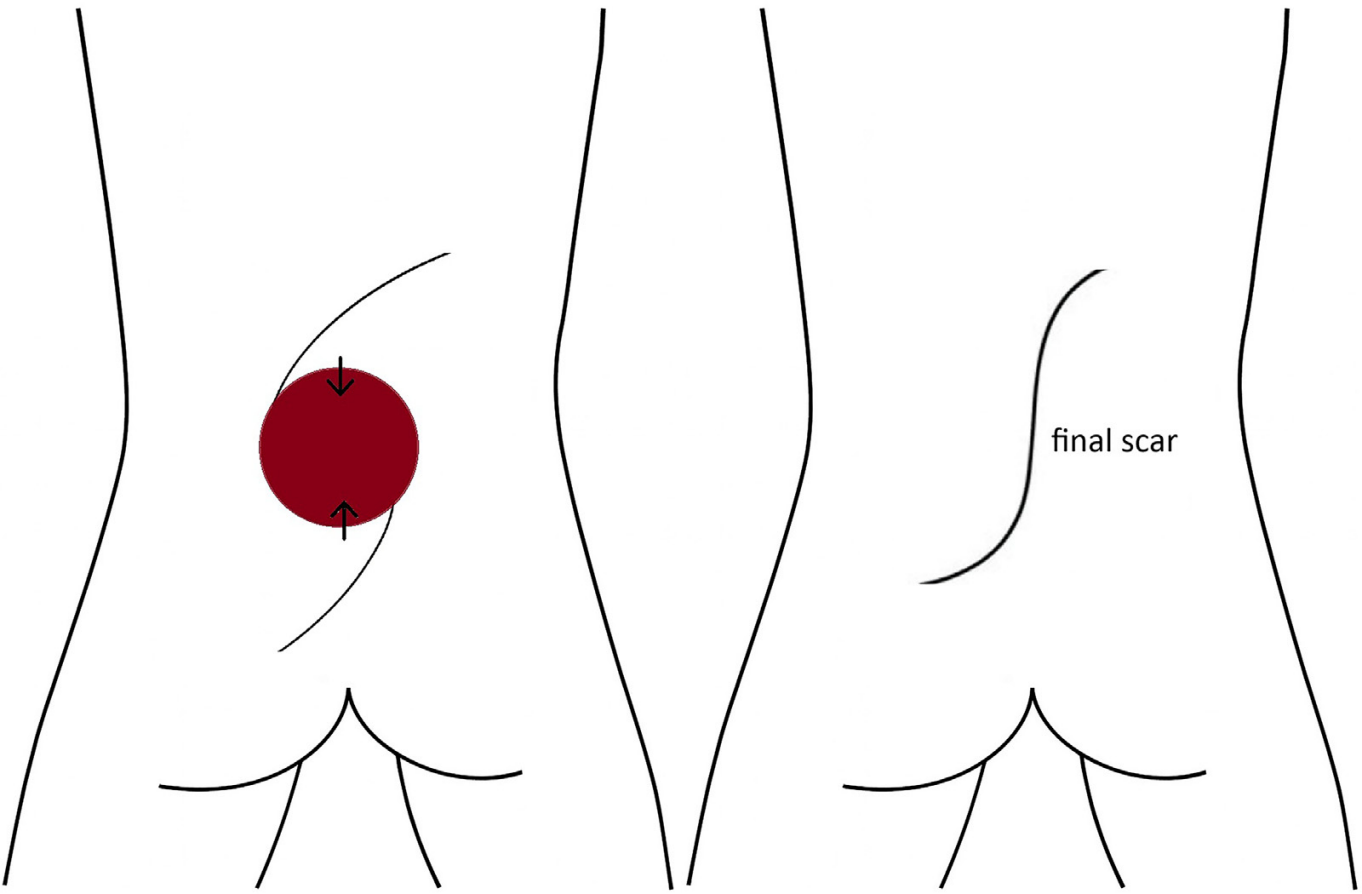
O-S flap schematic for myelomeningocele defect closure, showing transformation of a circular defect into an S-shaped scar through opposing arc advancement.

**Figure 7:**
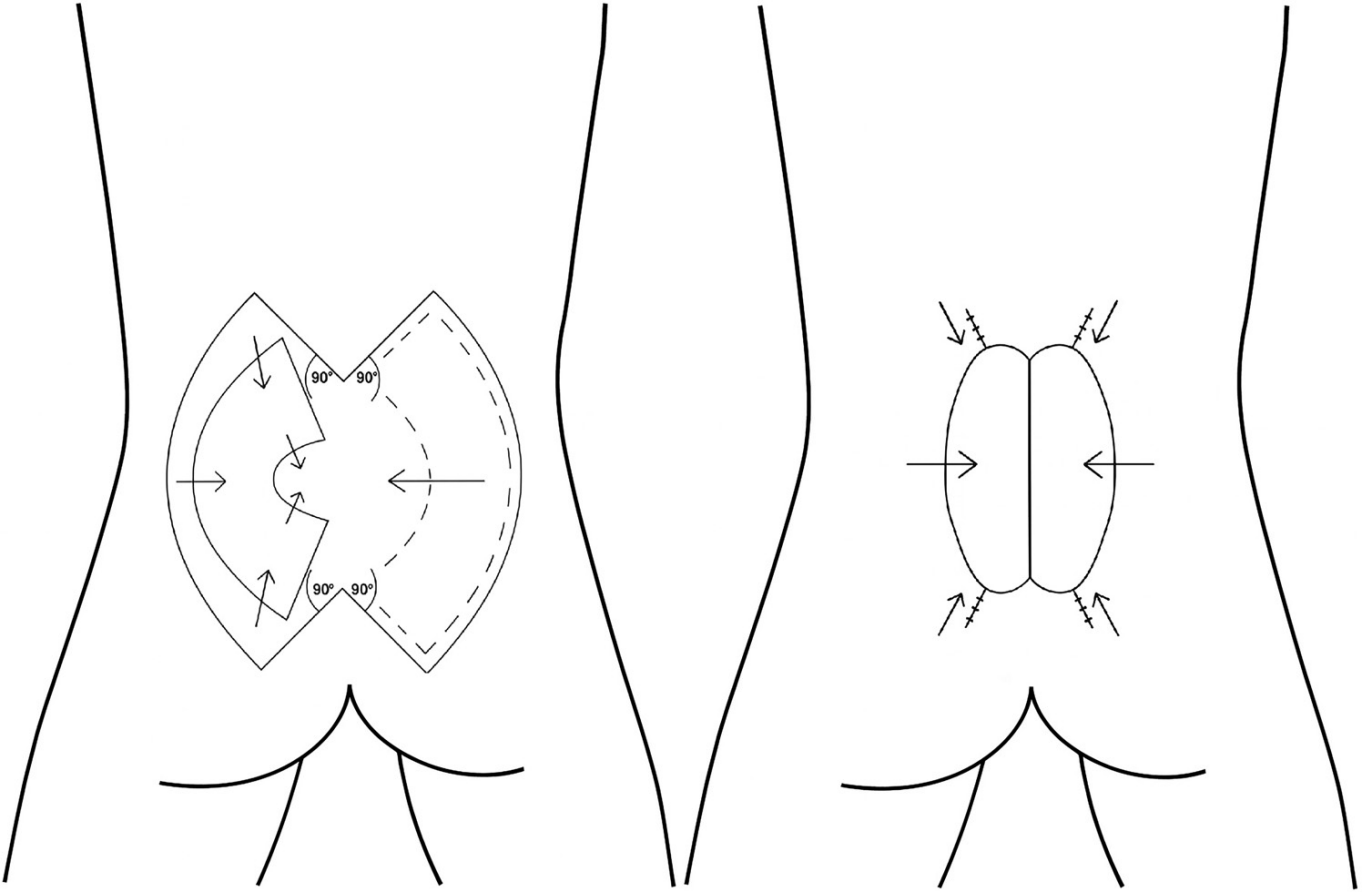
Bilateral keystone flap schematic for myelomeningocele defect closure, showing flap design and final result after advancement.

**Figure 8:**
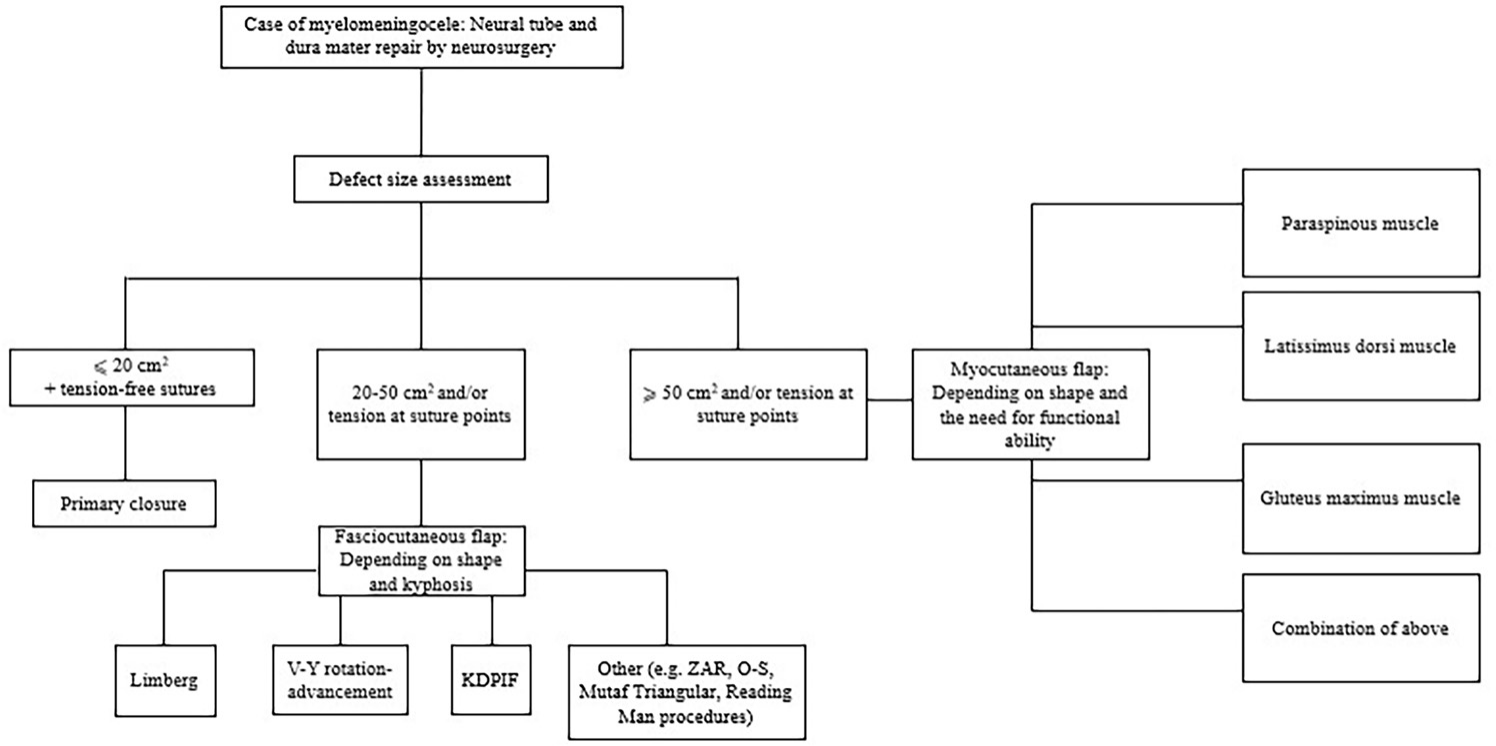
Suggested Algorithmic Approach for the Management of Myelomeningocele Defects.

Optimal management of myelomeningocele defects demands individualized surgical planning, prioritizing tension-free, durable closure. Primary closure is suitable for small, uncomplicated defects, whereas local fasciocutaneous flaps provide reliable outcomes for moderate-sized lesions. Large or complex defects may require musculocutaneous flaps despite higher surgical morbidity. An algorithmic, multidisciplinary approach ensures optimal outcomes tailored to patient-specific anatomical characteristics (Figure 1, Figure 2, Figure 3, Figure 4, Figure 5, Figure 6, Figure 7, Figure 8).

## Author contributions

EG: Conceptualization, Methodology, Formal analysis, Visualization, Writing, Original draft preparation. MZ: Data curation, Investigation, Writing, Original draft preparation. JS: Data curation, Investigation, Writing, Review and editing. KA: Formal analysis, Validation, Visualization, Writing, Review and editing. SBH: Conceptualization, Methodology, Project administration, Supervision, Validation, Writing, Review and editing.

## Ethical approval

Not required.

## Funding

This research did not receive any specific grant from funding agencies in the public, commercial, or not-for-profit sectors.

## Declaration of competing interest

The authors declare that there are no competing interests.
